# Clinicopathological pattern of oestrogen receptor, progesterone receptor and human epidermal growth factor receptor-2 over-expression of epithelial ovarian carcinomas in Nigeria

**DOI:** 10.4314/ahs.v23i3.29

**Published:** 2023-09

**Authors:** Mustapha Akanji Ajani, Aliyu Lawan, Temitope Oke, Galina Khramtsova, Ifeanyichukwu Nwanji, Ayodeji Salami, Olutosin Awolude, Henry Ebili, Michael E Onwukamuche, Elisabeth Sveen, Toshio Yoshimatsu, Olufunmilayo I Olopade

**Affiliations:** 1 Department of Pathology, University College Hospital, Ibadan. Nigeria; 2 Department of Medicine, University College Hospital, Ibadan. Nigeria; 3 Department of Medicine, Section of Haematology/Oncology, University of Chicago, Illinois. United States of America; 4 Department of Obstetrics and Gynaecology, University College Hospital, Ibadan. Nigeria; 5 Department of Morbid Anatomy and Histopathology, Olabisi Onabanjo University Teaching, Hospital, Sagamu, Nigeria; 6 Department of Histopathology, Nnamdi Azikiwe University Teaching Hospital, Nnewi. Nigeria

**Keywords:** Oestrogen receptor, Progesterone receptor, HER-2 over-expression, Epithelial ovarian cancer

## Abstract

**Background:**

Ovarian cancer is the leading cause of death from all gynaecological malignancies. Only few biomarkers of epithelial ovarian cancer (EOC) prognosis have been studied so far among Nigerian patients.

**Objective:**

To determine the pattern of oestrogen receptor (ER), progesterone receptor (PR) and human epidermal growth factor receptor 2 (HER-2) expression in patients with EOC seen in Nigeria

**Materials and Methods:**

This was a retrospective multicentre study of 102 cases of epithelial ovarian cancers. Relevant clinical information was obtained from hospital-based records in the 3 participating centres. Tissue microarrays were constructed using representative tumour tissue and the ER, PR and HER2 immunohistochemical staining was carried out at the University of Chicago, United States of America.

**Results:**

Serous carcinomas predominated (71% of cases). ER positivity was observed in 31.4%, PR positivity in 21.5% and HER2/neu in 16.7% of tumours. Fifty-two percent of tumours were triple negative. Serous tumours were significantly associated with ER positivity (p=0.001). Mean patient age for EOC was 52.6 ± 13.1 years. There were no statistically significant associations between hormone receptor status and histological grade, FIGO staging or survival.

**Conclusion:**

Serous tumours were significantly associated with ER expression while non-serous tumours tended to be triple negative.

## Introduction

Ovarian cancer is the most lethal gynaecological malignancy 1. Globally, ovarian cancer affects 239,000 patients and causes 152,000 deaths every year 2. Most patients present at an advanced stage, often due to paucity of symptoms early in the course of disease, and poor screening efforts especially in developing countries. Ovarian cancer is a heterogeneous entity, and this has explained in part, the limitation of successful treatment.

Approximately 90% of ovarian cancers are epithelial in nature, and a major histological subset of these are high-grade serous ovarian cancers. Cytoreductive surgery and platinum-based chemotherapy, remain the main treatment modalities for epithelial ovarian cancers, and this has remained largely unchanged over the last 25 years. Metastatic/recurrent ovarian carcinoma therefore remains an enigma, despite surgical and medical treatment advances 3. For this reason, it is of utmost importance to explore potential molecular targets that may improve the survival of ovarian cancer patients.

A growing body of evidence suggests that steroidal hormones may influence the pathobiology of ovarian cancers. Two of the most significant steroid hormones in this respect are oestrogen and progesterone. Oestrogens are essential regulators of ovarian growth and differentiation and have been speculated to contribute to the initiation and promotion of ovarian carcinogenesis via their interactions with their receptor, the oestrogen receptor (ER) on epithelial ovarian cancers (EOC) 4.

With respect to Progesterone relationship to EOC, genetic mutations such as loss of heterozygosity at 11q23.3-24.3 region (which contains the PR gene) has shown association with increased risk for ovarian cancer and poorer prognosis 4. Studies have shown that ER and PR are significantly expressed in ovarian cancers buttressing the theory that both hormones may play a role in ovarian carcinogenesis 5,6. In addition, there may be variations in ER and PR expression among the various subgroups of EOC 7.

Overexpression of HER2/neu has been documented in about 20-30% of EOC, with a number of studies suggesting that this overexpression is indicative of poorer prognosis 8,9. Conversely, it is reasonable to postulate that such tumours may benefit from targeted treatment with antibodies to HER2/neu.

Triple negative epithelial ovarian cancer is used to define a subset of EOCs without ER, PR and HER2 expression, as used in breast cancers. These tumours have been demonstrated to be more aggressive when compared to their non- triple negative epithelial ovarian cancer (non-TNEOC) counterparts 10.

In this multicenter study conducted in Nigeria, we sought to assess the prognostic and predictive effects of ER/PR and HER2 expressions on EOC prognosis, as well as their correlations and associations with histologic subtype survival.

## Materials and methods

### Patients and samples

A total of 102 Nigerian women diagnosed with primary epithelial ovarian cancer (EOC) at the Department of Pathology, University College Hospital, Ibadan; Morbid Anatomy and Histopathology Department, Olabisi Onabanjo University Teaching, Hospital, Sagamu; Department of Histopathology, Federal Teaching Hospital, Gombe and Department of Histopathology, Nnamdi Azikiwe University Teaching Hospital, Nnewi between 2005 and 2015, were evaluated retrospectively.

The formalin-fixed paraffin-embedded (FFPE) tissue blocks were constructed into tissue microarrays and subsequently stained with haematoxylin and eosin stains, ER, PR and HER2/neu immunostains at the University of Chicago, United States of America following standard procedures as previously described in a comparative study done by Sieh et al. across United States of America, Canada and Australia 7. Clinical data, including age at diagnosis, date of diagnosis, date of death or last clinic follow-up, and International Federation of Gynecology and Obstetrics (FIGO) stage were obtained. The FIGO stage at diagnosis was categorized as early (FIGO stages I and II), or advanced (FIGO stages III and IV). Histological subtypes of EOC were assessed on FFPE tissue blocks by using 2014 World Health Organization (WHO) classification of ovarian neoplasms 3. Histological grading (three-grade system) of Shimizu and Silverberg was used, which assesses architectural pattern, nuclear pleomorphism and mitotic activity 3.

All patients with a histological diagnosis of EOC were treated with cisplatin-based chemotherapy and were followed-up, up to December 2015. Overall survival (OS) is defined as the time from diagnosis to the last clinic visit or death

### Tissue microarray construction

A tissue microarray (TMA) was designed after selecting the most representative areas by experienced pathologists. There were 2 areas of interest corresponding to the most representative areas of the tumour blocks that were marked. Two areas were selected from each of the cases for tissue microarray construction. These marks guided the punch size of 1.0mm which was performed using a TMA instrument (Beecher Manual Tissue Arrayer). Tissue microarrays were constructed from FFPE tissue tumour block and adjacent histologic normal epithelium that served as an internal positive control.

### Assessment of immunohistochemical (IHC) analysis

All slides were independently assessed by three experienced pathologists blinded to the clinicopathological parameters of the disease, and cases with discordant scores were re-evaluated to obtain a consensus score. Two sets of tissue microarrays of each tumour were used for each marker. If the scores were different in the analyses, the higher staining score was considered. Nuclear immunohistochemical staining was considered for ER and PR while membrane IHC staining was considered for HER2/neu expression. Grading of nuclear ER and PR staining was performed using an immunoreactive H-scoring system obtained by the product of intensity of immunostaining as negative (0% tumour cell nuclei), weak (1-25%=1+), moderate (26-50%=2+) and strong (>50%=3+). Samples scored ≥1% tumour cell nuclei were considered positive. HER2/neu was scored visually according to the American Society of Clinical Oncology/College of American Pathologists (ASCO/CAP) 2007 guidelines [0: No staining or incomplete membrane staining which is faint or barely perceptible, and within ≤ 10% of the tumour cells; 1+: Incomplete membrane staining which is faint or barely perceptible, and within > 10% of the tumour cells; 2+: Circumferential membrane staining which is incomplete and/or weak or moderate, and within >10% of the tumour cells; or complete and circumferential membrane staining which is intense and within ≤ 10% of the tumour cells and 3+: Circumferential membrane staining which is complete and intense, and within > 10% of the tumour cells]. Samples scored as 0 or 1+ were considered to be negative while scores of 2+ and 3+ were considered to be positive.

### Statistical analysis

The data obtained were subjected to statistical analysis using IBM Statistical Product and Service Solutions (SPSS) version 23. Statistical analysis was used to evaluate statistical associations between expression of ER, PR and HER2/neu and clinicopathological parameters: age, FIGO stage, grade, and histological subtypes. The relationship between triple negative epithelial ovarian cancer (TNEOC) and clinicopathological parameters was also compared using Chi square. Overall survival analysis was determined using Kaplan-Meier method. Categorical variables were compared using the chi-square test. Statistical significance was defined as p <0.05.

### Ethical approval

This study was approved by the local ethics committee of the coordinating institutions in Ibadan, Sagamu, Gombe and Nnewi, and has been carried out in compliance with the guidelines of the Helsinki Declaration of 1975.

## Results

**Figure 1:**
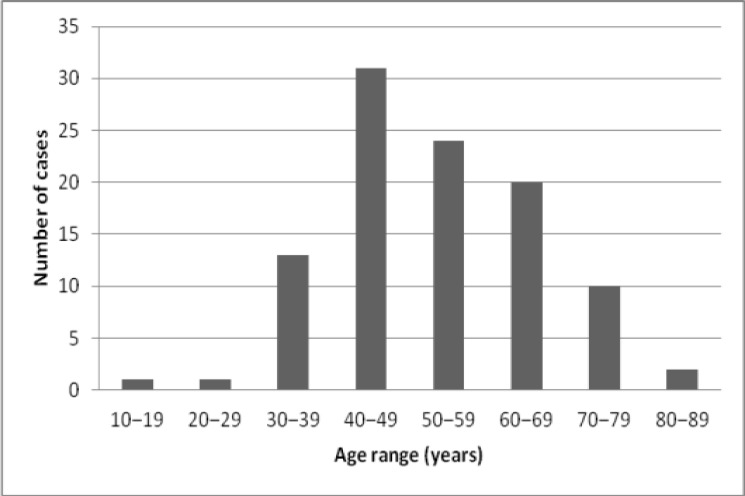
Age distribution of patients with histologically diagnosed epithelial ovarian cancer

One hundred and two cases of EOC met the inclusion criteria and were analysed. Briefly, mean patient age was 52.6 ± 13.1 years, while the peak age of occurrence of EOC was in the fifth decade of life. (Figure 1). Majority of patients (56%) had FIGO stage III–IV disease. Tumours were predominantly right sided (41.2%) with left sided tumours occurring in 34.3% of cases. Tumours were bilateral in 24.5% of cases.

**Figure 2:**
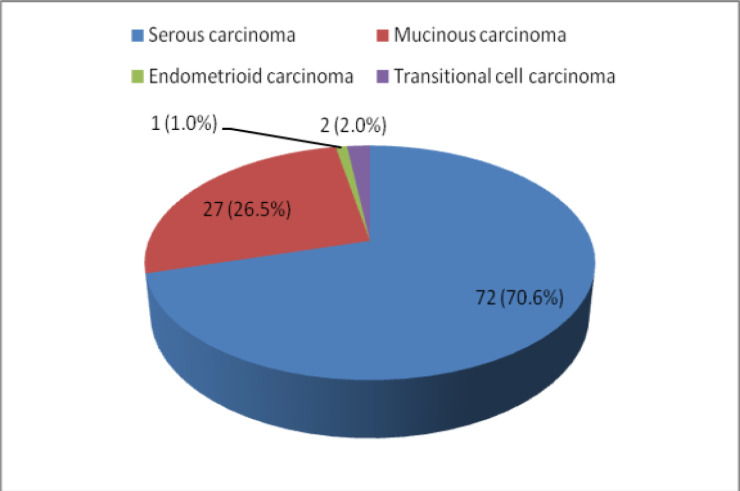
Histological subtypes of epithelial ovarian cancers

**Figure 3:**
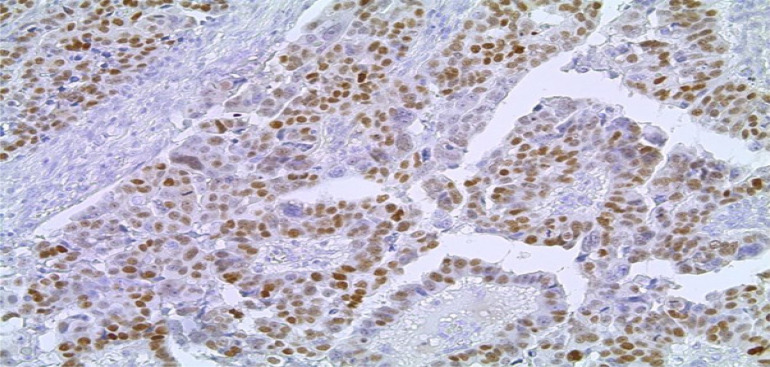
Photomicrograph showing strong nuclear staining for Oestrogen Receptor. (Immunohistochemistry, X400)

**Figure 4:**
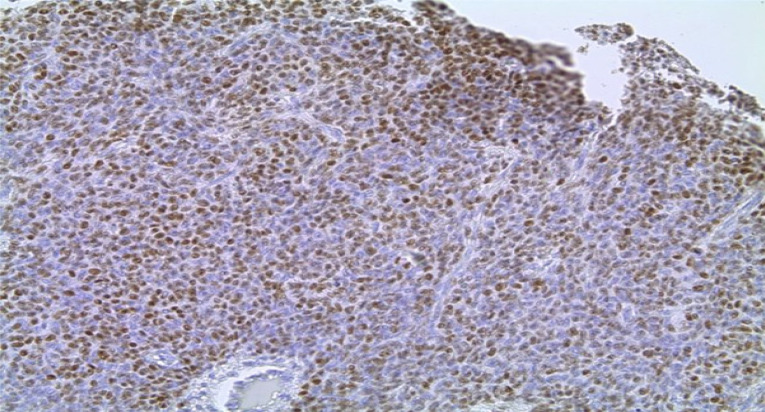
Photomicrograph showing strong nuclear staining for Oestrogen Receptor. (Immunohistochemistry, X400)

**Figure 5:**
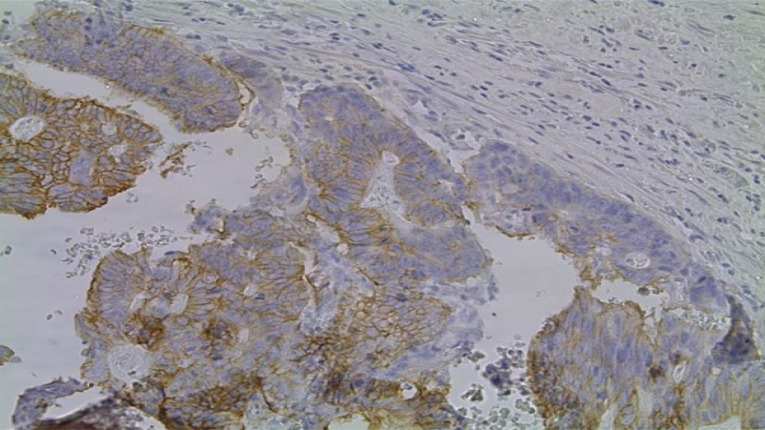
Photomicrograph showing strong membranous staining for HER-2/neu (Immunohistochemistry, X400)

Serous carcinomas constituted the predominant group, accounting for 71% (72/102) of cases, followed by mucinous 26% (27/102), transitional 2% (2/102) and endometrioid 1% (1/102) [Figure 2]. Across the spectrum of tumours, ER positivity was observed in 32 cases (31.4%), PR positivity in 21.5% and 16.7% of tumours were positive for HER2/neu (Figures3,4 and 5). 52% of tumours were triple negative.

Most patients with serous carcinoma had advanced stage cancers (68%) and poorly differentiated tumours (58.3%). Serous tumours were also significantly associated with ER positivity (p=0.001) [Table 1] when compared with other histological subtypes; other histological subtypes were more likely to be triple negative (p=0.014). There were no statistically significant associations between hormone receptor status and histological grade and FIGO staging.

**Table 1:** Relationship between FIGO stage and histological subtypes (P=0.001)

Histological subtypes		FIGO Stage			Total (%)

	I	II	III	IV	
Serous carcinoma	2	21	31	18	72 (71.0)
Mucinous carcinoma	3	14	3	7	27 (26.0)
Endometrioid carcinoma	1	0	0	0	1(1.0)
Transitional cell carcinoma	0	2	0	0	2 (2.0)
**Total (%)**	**6 (5.9)**	**37(36.3)**	**34 (33.3)**	**25(24.5)**	**102 (100)**

The mean overall survival duration of patients with EOC was 7.6 months from diagnosis [Table 2]. Mortality rate at 2 years was 100%. There was no significant association between hormone receptor status and survival. In addition, none of the key epidemiological and clinical features investigated for predicted survival duration.

**Table 2:** Survival rates of patients based on clinicopathological parameters

		Survival rate (%)			
Variable	≤ 6 months	7 - 12 months	13 - 18 months	19 - 24 months	> 24 months
Overall	43.1	20.6	10.8	6.9	0

**Age (year)**					
< 50	39.1	13.0	6.5	2.2	0
> 50	46.4	26.8	14.3	10.7	0
**Diagnosis**					
Serous	48.6	25.0	12.5	8.3	0
Mucinous	33.3	11.1	7.4	3.7	0
Endometrioid	0	-	-	-	-
Transitional	-	0	-	-	-
**Site**					
Bilateral	34.3	20.0	11.4	8.6	0
Left	52.0	28.0	20.0	12.0	0
Right	45.2	16.7	4.8	2.4	0
**Grade**					
1	40.0	13.3	6.7	0	0
2	45.5	27.3	13.6	11.4	0
3	41.9	16.3	9.3	4.7	0
**Stage**					
I	16.7	0	0	0	0
II	48.6	29.7	10.8	5.4	0
III	47.1	23.5	17.6	11.8	0
IV	36.0	8.0	4.0	-	0
**ER**					
Positive	53.1	31.3	15.6	12.5	0
Negative	38.6	15.7	8.6	4.3	0
**PR**					
Positive	45.5	18.2	9.1	4.5	0
Negative	42.5	21.3	11.3	7.5	0
**HER2/neu**					
Positive	41.2	11.8	-	-	0
Negative	43.5	22.4	10.6	5.9	0
**TNEOC**					
Tneoc	39.6	15.1	7.5	1.9	0
Non-Tneoc	46.9	26.5	14.3	12.2	0

TNEOC: Triple negative epithelial ovarian carcinoma

## Discussion

To the best of the authors' knowledge, this is the first, multicentre study in Nigeria detailing the associations between steroid receptor expression, HER2 status and important EOC clinic-pathological features. The expression rate of ER, PR and HER-2/ neu in this study was 31.4, 21.6% and 16.7% respectively. The figures obtained from other studies have ranged from 35 to 73.2% for ER and 15 to 69 for PR receptors. HER2/neu positive expression in this study was 16.7%, compared to figures ranging from 6.6% to 50% in other studies. HER2/neu over-expression in this study corroborated with what was found by Demir et al. and De Toledo et al 11,12, [See Table 3].

**Table 3:** Comparison of ER, PR and HER2/*neu* expressions in various studies

Author, year of publication	Location of study	Number of cases	Method	ER+ (%)	PR+ (%)	HER2+ (%)	Tnec (%)
Kommoss et al 1992 (14)	Germany	87	Immunohistochemistry	38	31	-	-
Ayadi et al 2010(15)	Tunisia	57	Immunohistochemistry	35.1	33.3	-	-
Tuefferd et al 2007(16)	France	320	Immunohistochemistry with FISH	-	-	6.6	-
Slamon et al 1989(17)	USA	72	Immunohistochemistry	-	-	50	-
Liu et al 2009 (10)	China	116	Immunohistochemistry	71.6	53.4	22.4	15.5
Demir et al.2014(11)	USA	82	Immunohistochemistry	73.2	51.2	18.3	18.3
De Toledo et al 2014 (12)	Brazil	152	Immunohistochemistry	46.7	31.6	12.5	39.5
**Present study (2015)**	**Nigeria**	**102**	**Immunohistochemistry**	**31.4**	**21.6**	**16.7**	**52**

While the relatively low prevalence of hormone receptor positivity in this study suggests that Nigerian patients with ovarian cancer may benefit less from hormone receptor antagonists, further studies utilizing large patient cohorts may be required. In particular, serous carcinomas may deserve more attention as they constituted the majority of cancers and demonstrated significant association with ER overexpression. Non serous tumours were more likely to be triple negative, corroborating a different pathway of malignant transformation in this group of tumours.

Overall survival rate and time was particularly low in this study, highlighting the significant burden ovarian cancers pose in Sub-Saharan Africa as compared to what is obtainable elsewhere 13. The results of the study did not reveal a significant relationship between the hormone receptor status and survival of patients with EOC. Conversely, a meta-analysis assessed the prognostic effect of hormone receptors in EOC and concluded that, PR expression was significantly associated with better overall survival (OS) and progression-free survival (PFS) times. These might not be unconnected with absence of receptor isoforms such as ER-α, and ER-b in our study. In a similar vein, we did not find any significant association between triple negative status and survival status in our study. In contrast, a few studies have demonstrated that these tumours are significantly associated with shorter progression free survival and overall survival times compared to the non-triple negative subtype 14,15,16,17.

Perhaps more telling is that this study failed to reveal any potential clinic-pathological predictors of survival among patients with EOC, suggesting that systemic factors such as low socioeconomic status and lack of access to standard care may be at play in determining survival, as opposed to well established factors such as tumour stage and grading.

## Conclusion

The peak age of occurrence of epithelial ovarian cancer was in the fifth decade of life. Serous carcinomas were the most common histological subtype of epithelial ovarian cancer with majority of patients presenting with advanced stage disease.

Serous tumours were significantly associated with ER expression while non-serous tumours tended to be triple negative. There was no significant association between overall survival and clinic-pathological parameters, including hormone receptor status in this study.
